# A Revival in Antiseptics

**Published:** 1916-01-08

**Authors:** 


### A REVIVAL IN ANTISEPTICS.

### Two Interesting Papers.

The hospital world is bound to be interested in what is almost a revival in the use of antiseptics. The enormous number of septic wounds now treated in hospitals has forced upon surgeons the reconsideration of those antiseptic solutions which, at one time so much in vogue, had largely disappeared from surgical practice. This return to the use of solutions of antiseptic repute is not being made without inquiry into the solidity of the claims formerly made for them, and familiar disinfectants are being tried in new ways. Only recently we drew attention to an article in the "Medical Annual" for last year in which reference was made by Surgeon-General G. Lenthal Cheatle to the efficient antiseptic properties of an alcoholic solution of perchloride of mercury with malachite green. Since the date of this preliminary report his collaborators, Dr. Paul Fildes and Dr. Rajchman, have made further important experiments, and have published a practical paper on the subject in the *Lancet*. The matter is one which must affect the majority of hospitals very shortly, and one which will have considerable effect on their expenditure and on the rapidity with which this septic class of wounds can be made to heal. Consequently, if the claims made for this combination of antiseptics are substantiated, soldiers with chronic suppurating wounds will not be so long lost to the Service, and a certain number of beds will be set free earlier.

The question of the expense of the mixture is not discussed by the authors of the paper alluded to, and it is to be hoped that some information on this point will shortly be forthcoming. As the solution is applied by means of a spray, there should be economy as compared with the usual lavish use of solutions. The ingredients of the mixture are given as follows:—Two solutions are made separately and mixed in equal parts as required. One consists of a 1 in 100 solution of perchloride of mercury crystals in 80 per cent. industrial spirit; the other of a solution of *pure* malachite green of the same strength in the same medium. Our previous ideas regarding the properties of corrosive sublimate receive something of a shock at the proposal to apply this substance, even in a spray, in so high a strength as 1 in 200, but the results obtained by the authors appear to demonstrate the superiority and efficiency of this method.

Contemporary with this paper there appeared an article in the *British Medical Journal* relating to experimental observations on the antiseptic action of hypochlorous acid. This acid is simply and readily produced, either in the gaseous state or in solution, by the interaction of bleaching powder and boric acid. The merits of this simple method of obtaining an apparently powerful antiseptic from two easily procurable substances are sure to meet with speedy consideration.

